# Yellow Fever

**Published:** 1903-09

**Authors:** 


### YELLOW FEVER.

---

Yellow fever is knocking at the door of Texas. Dr. Tabor says that there is an epidemic of "fever" at Neuvo Laredo, just across the river from Laredo, which the native physicians there call "dengue," but he is suspicious and has taken the same precautions as if it were a pronounced type of yellow fever, which, no doubt, it is. Dr. Tabor is positive of one or more cases of yellow fever at Nuevo Laredo. He has issued the following instructions to county health officers:

*"To County and City Physicians:*

"On account of yellow fever existing at several places in Mexico in cities near Texas, and wishing to prevent its introduction into this State, I desire to request that you take up at once with the proper authorities the matter of sanitation in your communities. I especially urge that means be adopted for the destruction of mosquitoes, as these insects are now known to be distributors of yellow fever. The expense for the total extermination of mosquitoes would be very small, and the means are very simple.

"The free use of kerosene oil in tanks, cisterns and pools of stagnant water or in sewers will effect the destruction of these insects. The oil should be used twice a week. An ounce of refined oil put into a cistern will destroy all wiggletails and mosquitoes and will not affect the water. Crude oil could be used in other places.

"In addition to these methods for the destruction of mosquitoes other measures should be adopted for the purpose of putting your cities and towns in thorough sanitary condition by a general cleaning up and burning of all trash and refuse matter and free use of disinfectants, such as lime, carbolic acid, bichloride of mercury and ashes. This should be done for the prevention of other diseases, as well as yellow fever.

"As yellow fever is near us, having made its appearance at the border, I respectfully request that this be given your prompt attention, and that you take the matter up with the proper authorities and secure also the co-operation of all your citizens.

"Prompt and vigorous action may prevent a disastrous epidemic. Please impress upon your officials the necessity of clothing you with proper authority and giving you financial assistance to put these measures into immediate effect. The delay of a day may prove disastrous.

"Earnestly soliciting the prompt co-operation of every health official in the State, I remain,

"Your obedient servant,

(Signed) "George R. Tabor,

"State Health Officer."

